# Interruption of enteral tube feeding during chest physiotherapy in critically ill adults: A scoping review

**DOI:** 10.2478/jccm-2026-0002

**Published:** 2026-01-30

**Authors:** Ruvistay Gutierrez-Arias, Francisco Salinas-Barahona, Pamela Seron

**Affiliations:** Departamento de Apoyo en Rehabilitación Cardiopulmonar Integral, Instituto Nacional del Tórax, Santiago, Chile; INTRehab Research Group, Instituto Nacional del Tórax, Santiago, Chile; Exercise and Rehabilitation Sciences Institute, School of Physical Therapy, Faculty of Rehabilitation Sciences, Universidad Andres Bello, Santiago, Chile; Departamento de Apoyo en Rehabilitación Cardiopulmonar Integral, Instituto Nacional del Tórax, Santiago, Chile; INTRehab Research Group, Instituto Nacional del Tórax, Santiago, Chile; Escuela de Kinesiología, Universidad de Las Américas, Santiago, Chile; Departamento de Ciencias de la Rehabilitación, Facultad de Medicina, Universidad de La Frontera, Temuco, Chile; Centro de Excelencia CIGES, Facultad de Medicina, Universidad de La Frontera, Temuco, Chile

**Keywords:** chest physiotherapy, enteral tube feeding, critical care, safety, aspiration pneumonia

## Abstract

**Introduction:**

Numerous reports indicate that the nutritional targets of critically ill patients are frequently not met. In daily clinical practice, it is often recommended to temporarily stop enteral tube feeding in patients on mechanical ventilation (MV) who are undergoing chest physiotherapy. This is because adverse events can occur and potentially cause vomiting and increase the risk of aspiration pneumonia.

**Aim of the study:**

To identify, characterise, and analyse the available evidence on the interruption of enteral tube feeding in critically ill adult patients receiving MV before or during chest physiotherapy.

**Materials and Methods:**

We conducted a scoping review following the recommendations of the Joanna Briggs Institute. We conducted a systematic search of MEDLINE (Ovid), Embase (Ovid), CENTRAL (Cochrane Library), CINAHL (EBSCOhost), and other search resources until March 2025. We included studies of any design that addressed the application of chest physiotherapy in adults on MV and receiving enteral tube nutrition. Study selection and data extraction were performed in duplicate, and disagreements were resolved by consensus.

**Results:**

We include four studies that were published between 2018 and 2024. One observational study reported that enteral tube feeding was discontinued due to the application of chest physiotherapy in patients in prone and supine MV. In the other three studies, which contribute to a clinical practice guideline, discontinuation of enteral tube feeding is recommended 30 minutes before using the head-down position as a bronchial drainage manoeuvre. However, no studies report on the safety of chest physiotherapy when enteral tube feeding is either discontinued or continued.

**Conclusion:**

There is no empirical evidence to justify routinely stopping enteral tube feeding during chest physiotherapy in MV patients. Future primary studies should report on the management of enteral tube feeding before or during chest physiotherapy interventions, as well as document any adverse events that may occur during its application.

## Introduction

Nutritional therapy is essential for achieving positive clinical outcomes in critically ill patients [1,2,3]. Numerous studies have demonstrated that appropriate nutritional intervention is associated with decreased mortality rates, reduced duration of mechanical ventilation (MV), and shorter lengths of stay in the intensive care unit (ICU) [4,5,6,7]. In this context, various methods of nutritional delivery are employed during the initial stages of intensive care, with enteral tube feeding representing the most utilised approach [8]. However, numerous reports indicate that the nutritional target of critically ill patients is frequently not met [9]. This situation is attributable to factors such as intolerance to enteral nutrition and the recurrent interruption of nutritional therapy aimed at preventing regurgitation or emesis during diagnostic reasons, such as radiological procedures, and non-invasive ventilation dependency [10,11,12,13,14].

Chest physiotherapy is a frequently employed intervention in critically ill patients receiving MV [15,16]. This multimodal intervention, covering a broad spectrum of techniques [17]. This aims to enhance airway clearance and improve the ventilation-perfusion ratio by restoring lost gas exchange areas or favouring perfusion in well-ventilated regions [18]. Flow and pressure changes occur in the respiratory system to achieve these effects, prompted by manual manoeuvres applied to the thoracoabdominal compartment, therapeutic positions, and even with the assistance of MV. In this context, it is common to observe that in daily clinical practice, temporary interruption of enteral tube feeding may be advised in patients with MV undergoing chest physiotherapy [19], especially when the feeding tube is positioned in the stomach and not advanced through the pylorus [20]. This is because adverse safety events can occur due to external pressures on the thoracoabdominal cavity, potentially causing gastroesophageal reflux and increasing the risk of aspiration pneumonia.

We performed an initial search across PubMed, Google Scholar, The Cochrane Library, and Epistemonikos to identify any evidence syntheses that describe or support the strategy of prophylactically interrupting enteral tube feeding in patients undergoing MV who are receiving chest physiotherapy. However, to date, no scoping or systematic reviews have addressed this research area. Therefore, our study aims to identify, characterise, and analyse the available evidence on the interruption of enteral tube feeding in critically ill adult patients receiving MV before or during chest physiotherapy.

Our review aimed to answer the following questions:

- Are there primary studies that have assessed the impact of pre-emptively discontinuing enteral feeding on the safety of chest physiotherapy in critically ill adults receiving MV?
- What are the current recommendations on this practice?
- If studies on this topic have been conducted, how has feeding interruption been implemented, and what chest physiotherapy techniques have been utilised?

## Materials and methods

We conducted a scoping review according to the Joanna Briggs Institute manual [21]. Our review protocol was prospectively registered with the International Platform of Registered Systematic Review and Meta-Analysis Protocols (INPLASY) under the number INPLASY202380117. We report our study following the recommendations of the Preferred Reporting Items for Systematic Reviews and Meta-Analyses extension for Scoping Reviews (PRISMA-ScR) [22].

### Eligibility criteria

*Participants:* We included studies that enrolled at least one patient aged 15 years or older who underwent invasive MV via orotracheal or nasotracheal tube, or tracheostomy, and who received enteral nutrition therapy. Other sociodemographic variables, medical diagnoses, comorbidities, prior functional status, or duration of MV did not restrict study inclusion.

*Concept:* We included studies that evaluate the effects, effectiveness, efficacy, cost-effectiveness, and safety of chest physiotherapy. Our review examines both manual and instrumental techniques, regardless of their therapeutic objectives. Specifically, we consider postural drainage, chest percussion, vibration, thoracic oscillation, chest shaking, huffing, directed coughing, thoracic expansion, forced exhalation or expiration techniques, manual hyperinflation, and other reported techniques.

*Context:* We include studies conducted wholly or partially in the ICU, intermediate care, postoperative recovery, or emergency units.

*Resource type:* We included experimental, quasi-experimental, and descriptive or analytical observational studies. These could be retrospective, prospective, cross-sectional, or mixed. The presence of control groups was not a limiting factor for study inclusion. We accepted both multi-centre and single-centre studies conducted in any country. Additionally, we incorporate secondary studies, including narrative reviews, clinical practice guidelines, and society statements or expert consensus.

### Search strategy

Our search strategy comprised a three-step process. First, we searched PubMed, Google Scholar, the Cochrane Library, and Epistemonikos to identify the “seed references”. These references needed to include keywords for at least two of the three concepts: “enteral tube feeding”, “chest physiotherapy”, and “intensive care”. Subsequently, using the “seed references”, we identified the most repeated words and concepts using the Word Frequency Analyzer tool from The Evidence Review Accelerator (TERA) [23]. We accepted sentences of up to three words and limited concept identification to at least three occurrences. These concepts were also complemented with suggestions provided by ChatGPT^®^.

In the second step, we developed the search strategy for MEDLINE (Ovid) based on concepts identified in the initial stage. This strategy was created by a researcher specialising in systematic searches and reviewed by a clinician specialising in respiratory therapy in critical care. Once consensus was reached on the strategy for MEDLINE (Ovid), it was adapted for Embase (Ovid), CENTRAL (Cochrane Library), and CINAHL (EBSCOhost) (Supplementary Tables S1-S4).

In the final step, we used search terms like those employed in scientific databases to search grey literature in Google Scholar. We reviewed the first 300 records ordered by relevance [24] and conducted a secondary search of expert consensus on recommendations for the performance of chest physiotherapy in MV patients (Supplementary Table S5). Furthermore, we performed backward and forward citation searches for the studies that met our eligibility criteria. To achieve this, we utilised the Citationchaser^®^ web tool [25].

The last date we conducted our search across all databases was March 21, 2025.

### Study selection

After finishing the search, we removed duplicate records with TERA’s Deduplicator tool [26]. We reviewed all studies in duplicate. Initially, we review the titles and abstracts of the records and rate them as either included or excluded. Records rated as included by at least one reviewer proceed to the full-text reading phase. In this stage, we verify that the manuscripts meet all eligibility criteria. We resolve any disagreements through consensus or by involving a third reviewer. We utilise the Rayyan web application to screen by title, abstract, and full-text [27].

### Data extraction

We developed an extraction form specific to our review in a Microsoft Excel^®^ spreadsheet. When available, we retrieved bibliometric information on the studies (title, journal, year of publication, authors), methodology (study objective and design, inclusion and exclusion criteria for participants, outcomes assessed), a description of the strategy or methods for discontinuing enteral tube feeding during chest physiotherapy (timing, requirements, indications), and adverse events reported during chest physiotherapy. Regarding secondary studies, the clinical practice recommendations were extracted, and the sources of information supporting these guidelines were identified.

We conducted the data extraction in duplicate. Two reviewers tested the data extraction form with two studies. Subsequently, we made the necessary adjustments to the form to enhance its reproducibility. Any disagreements were resolved by consensus or through a third reviewer.

### Data analysis and synthesis

We detail the study selection process using a PRISMA flowchart and provide the reasons for excluding the reviewed studies in full-text. We report the first eligibility criterion not met in the following order: participants, concept, context, and resource type.

When possible, we describe the strategies utilised to discontinue enteral feeding for chest physiotherapy as outlined in the studies. Furthermore, we provided a list of recommendations for clinical practice, as noted in the secondary studies, and linked them to the primary source. Additionally, we report the incidence of adverse events mentioned in the included primary studies. Our findings are presented in narrative form.

## Results

### Search results

**Fig. 1.**
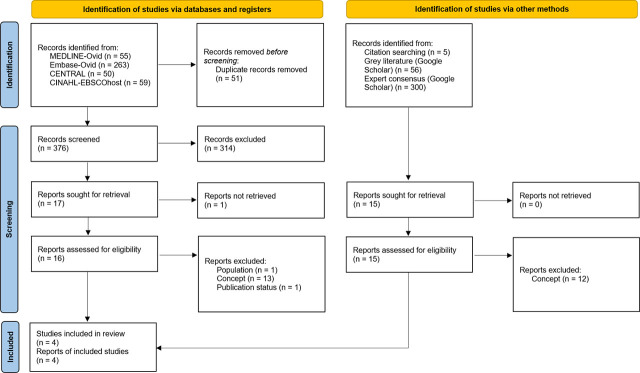
PRISMA study selection flow diagram

Our search strategy in scientific databases identified 376 unique records, of which 314 were deemed irrelevant. Of the 16 studies reviewed in full-text, we excluded 15, with the most common reason being “Concept” (Supplementary Table S6). In our exploration of other resources, we reviewed 15 full-text studies, of which three met our eligibility criteria. Altogether, our scoping review included four studies [28,29,30,31] (Figure 1).

### Characteristics of included studies

The four studies were published between 2018 and 2024 and were conducted in India [28] and Australia [29,30,31]. One of them aimed to assess the feasibility and tolerability of enteral tube feeding in prone MV patients [28]. The remaining three were conducted by the same core group of researchers, who detailed the process of developing a clinical practice guideline of recommendations for the respiratory physiotherapy assessment and treatment of adult patients on MV due to community-acquired pneumonia (Table 1) [29,30,31].

**Table 1.** Characteristics of included studies

**Id**	**Design**	**Objective**	**Inclusion and exclusion criteria**	**Outcomes assessed**
Savio 2020 [28]	Prospective observational study	To assess the feasibility, tolerance, and effectiveness of enteral nutrition in critically ill patients receiving invasive MV in the prone position for severe ARDS	Inclusion: Adult patients with ARDS who underwent invasive MV in the prone position.	Feasibility and tolerance of enteral nasogastric feeding
			Exclusion: Patients who were not fed enterally due to significant hemodynamic instability	

van der Lee 2019 [29]	Expert consensus (modified Delphi technique)	To determine an expert consensus for respiratory physiotherapy management of intubated and mechanically ventilated adults with community-acquired pneumonia	Inclusion: minimum of 5 years as a qualified physiotherapist and a minimum of 3 years of experience in critical care and either (a) met the standards for qualification of “specialist” in cardiorespiratory physiotherapy within the Australian Physiotherapy Association or (b) had a minimum of 2 years of experience working in a senior position within critical care or a minimum of five publications on critical care physiotherapy (including coauthor)	Consensus statements for respiratory physiotherapy in adults with community-acquired pneumonia on MV. The statements were grouped into Physiotherapy assessment, Physiotherapy treatment, 2.1) Positioning, 2.2) Hyperinflation techniques, 2.3) Manual chest wall techniques, 2.4) Normal saline instillation, and 2.5) Active modes of treatment and mobilisation

van der Lee 2020 [30]	Qualitative mixed methods study	To conduct a peer review of the expert consensus statements for respiratory physiotherapy management of community-acquired pneumonia to determine their acceptability to Australian multidisciplinary ICU staff and to explore what adaptations might be required to enable them to be developed into a relevant and useful guideline for clinical practice	Senior medical, nursing and physiotherapy clinicians working in an Australian Level 2 or 3 ICU	Clinical validity and applicability of the expert consensus statements (van der Lee 2019). New factors perceived by participants to influence the application of the consensus statements into clinical practice

van der Lee 2024 [31]	Clinical practice guideline	To develop a clinical practice guideline for physiotherapy management of adults invasively ventilated with community-acquired pneumonia using the best available evidence	Not applicable	Twenty-six recommendations for clinical physiotherapy practice

ARDS: Acute respiratory distress syndrome; ICU: Intensive care unit; MV: Mechanical ventilation.

### Interruption of enteral tube feeding

In the study by Savio *et al.* (2020), it is noted that enteral tube feeding was interrupted in both prone and supine positions due to chest physiotherapy [28]. However, the timing of the interruption is not specified, and it is not clear whether the feeding pump was always stopped during chest physiotherapy. Additionally, it was not specified whether the reported adverse events were directly related to the application of chest physiotherapy [28]. In the case of the studies by van der Lee *et al.* [29,30,31], it is recommended that enteral tube feeding be stopped 30 minutes before head-down positioning as part of bronchial drainage techniques (Table 2).

**Table 2.** Strategy for discontinuing enteral tube feeding during chest physiotherapy

**Id**	**Strategy**	**Timing**	**Adverse events**	**Limitations**
Savio 2020 [28]	Interruption of enteral feeding during chest physiotherapy in prone or supine MV patients	Chest physiotherapy was counted as a procedure in the ICU. Interruption of enteral feeding due to ICU procedures accounted for 72% of the total time in prone patients and 41% in supine patients	No patient experienced vomiting or nausea while in the prone position, but there were 12 instances of vomiting or nausea in the supine position.	The study fails to establish a direct connection between vomiting events and the administration of chest physiotherapy
van der Lee 2019 [29]	If the head-down tilt position is used to minimise the risk of aspiration of gastric contents into the lungs, it is best to either stop enteral feeds or ensure the stomach is emptied by aspirating the nasogastric tube	For at least 30 minutes before positioning	Not applicable	Not applicable
van der Lee 2020 [30]	If the head-down tilt position is used to minimise the risk of aspiration of gastric contents into the lungs, it is best to either stop enteral feeds or ensure the stomach is emptied by aspirating the nasogastric tube	For at least 30 minutes before positioning	Not applicable	Not applicable
van der Lee 2024 [31]	If the head-down tilt position is used, the enteral feed should either be withheld or the nasogastric tube aspirated to empty the stomach, to minimise the risk of aspiration of gastric contents into the lungs (Conditional recommendation; GRADE certainty of evidence: Very low)	For at least 30 minutes before positioning	Not applicable	Not applicable

**ICU:** Intensive care unit; **MV:** Mechanical ventilation.

## Discussion

Four studies published between 2018 and 2024 met the eligibility criteria of our scoping review. One study was observational [28], and the other three were an expert consensus [29], expert consensus validation [30], and a clinical practice guideline [31] conducted by the same core group of researchers. While these studies report that enteral tube feeding is discontinued during chest physiotherapy and recommend discontinuing enteral tube feeding when performing chest physiotherapy in a specific position, there is no empirical data to support routine discontinuation of enteral tube feeding during this procedure. Furthermore, there is no data on the safety of chest physiotherapy regarding the risk of aspiration pneumonia due to potential gastroesophageal reflux while MV patients continue to receive enteral tube feeding. Based on the above, our scoping review identified a knowledge gap that must be addressed.

Contrary to a recently published meta-analysis [32], Savio *et al.* (2020) concluded that enteral tube feeding in prone MV patients is feasible and as well tolerated as in supine patients. Furthermore, this study reports that the duration and causes of enteral feeding interruption were similar between prone and supine patients, including chest physiotherapy. However, no details are provided on strategies or protocols for interrupting enteral tube feeding before or during chest physiotherapy [28]. On the other hand, although adverse events such as vomiting or nausea are reported, it is not possible to attribute them to chest physiotherapy [28].

Chest physiotherapy involves a wide range of techniques [33,34]. These are often applied to MV patients despite their uncertain effectiveness in relevant outcomes [35,36,37,38], and even in the face of unfavourable results [39]. Many chest physiotherapy techniques involve generating changes in chest pressures and airway flows to increase lung volumes and enhance clearance of secretions. In response to this, the discontinuation of enteral tube feeding has been proposed [19], as some chest physiotherapy techniques may undesirably generate regurgitation of stomach contents, increasing the risk of aspiration. In the three studies by van der Lee *et al.*, aimed at developing a clinical practice guideline for the management of respiratory physiotherapy in adults on MV due to community-acquired pneumonia, discontinuation of enteral tube feeding 30 minutes before using the head-down position is recommended [29,30,31]. Furthermore, it is specified that this position is contraindicated in patients with abdominal distension, vomiting, or poor feed absorption [31]. However, this recommendation is conditional as it is based on very low certainty of evidence. In addition, some of the intensivists interviewed in the expert consensus validation study expressed concern about not achieving caloric intake goals due to discontinuation of enteral tube feeding because of chest physiotherapy [30].

Some studies have reported that low caloric intake is associated with poor outcomes in critically ill adults [1,2]. Additionally, adequate protein intake is crucial for the effective physical rehabilitation of critically ill patients [40]. In this context, and given the lack of empirical data on its safety, it seems unjustified to routinely discontinue enteral tube feeding before or during chest physiotherapy. However, given the lack of description of enteral tube feeding management in clinical trials on chest physiotherapy [15,20], it is also not possible to establish that this intervention is safe in MV patients receiving continuous enteral feeding.

Future studies are needed to describe the protocols of enteral tube feeding management in MV patients, specifically when different chest physiotherapy techniques are applied. It is necessary to report more details on adverse events, their timing in relation to the application of chest physiotherapy, and stratified according to the type of technique used. Additionally, the daily practices and beliefs of physiotherapists on this topic should be studied. On the other hand, new studies that intend to include chest physiotherapy as a primary intervention or co-intervention should report in detail the management of enteral tube feeding and the endotracheal tubes or tracheostomies used, as well as their management, since not all have been shown to seal the airway adequately [41,42].

This study has potential limitations. Our search strategy for electronic databases may have missed some studies that met our eligibility criteria, as it was specific in considering terms for ‘enteral feeding’. However, we also conducted hand and grey literature searches for studies of any methodological design, including expert consensus and clinical practice guidelines. In addition, we reviewed the studies included in systematic reviews that evaluated the effectiveness of chest physiotherapy in adults in MV, showing that none of them addressed the management of enteral tube feeding. Another aspect that could be considered a limitation of the evidence is that the same group of researchers conducted three of the four studies included in our review. The recommendation to discontinue enteral tube feeding is not based on empirical studies, meaning the experts’ judgments in these studies may not be entirely generalized.

## Conclusion

There is no empirical evidence to justify routinely stopping enteral tube feeding during chest physiotherapy in MV patients. The recommendations existing in the literature are specific to the head-down position and are based on very low certainty of evidence. Additionally, there are no reports of strategies used to interrupt enteral tube feeding during chest physiotherapy. Based on the above, our scoping review identified a knowledge gap that must be addressed.

Future research should investigate ICU protocols, daily practices, and physiotherapists’ perceptions regarding enteral tube feeding management when implementing interventions like chest physiotherapy.

Furthermore, new primary studies, regardless of their methodological design, should report on the management of enteral tube feeding before or during the chest physiotherapy intervention, as well as document any adverse events during its application. This would facilitate a better understanding of this field of study, enabling the generation of evidence-based recommendations in the future.
